# THE EFFECTS OF SLEEP DEFICIENCY ON MULTIMORBIDITY AMONG OLDER ADULTS IN THE PHILIPPINES AND VIETNAM

**DOI:** 10.1093/geroni/igac059.2415

**Published:** 2022-12-20

**Authors:** Tuo-Yu Chen, Grace Cruz, Nguyen Vu, Yasuhiko Saito

**Affiliations:** Taipei Medical University, Taipei City, Taipei, Taiwan (Republic of China); University of Philippines, Diliman, Quezon, Philippines; Institute of Population, Health and Development, Hanoi, Ha Noi, Vietnam; Nihon University, Chiyoda-ku, Tokyo, Japan

## Abstract

Multimorbidity increases the risks of disability and death and are prevalent among older adults. Evidence shows that sleep might play an important role in multimorbidity. However, previous research studies sleep quality and quantity in isolated manners, where in reality sleep quality and quantity are likely to affect each other. Besides, no study has been done to understand such relationship among older adults in low- and middle- income counties where the prevalence of multimorbidity increases rapidly. This study investigated the relationship between sleep deficiency (i.e., poor and insufficient sleep) and multimorbidity among community-dwelling older adults in the Philippines and Vietnam. Cross-sectional data were obtained from the Longitudinal Study of Ageing and Health in the Philippines (N = 3,562) and the Longitudinal Study of Ageing and Health in Vietnam (N = 3,936). Multimorbidity was defined by having two or more of chronic conditions (i.e., heart disease, heart attack, cardiovascular disease, hypertension, diabetes, lung diseases, renal diseases, liver diseases, and arthritis). Sleep deficiency was conceptualized as self-reported short sleep duration (< 6 hours), having trouble with falling asleep or maintaining sleep, and/or experiencing non-restorative sleep. Logistic regression was used to analyze the data adjusting for demographics, body mass index, sleep medications, naps, mental health, and lifestyle. The results showed that having deficient sleep was significantly related to increased odds of experiencing multimorbidity by about 81% in the Philippines and Vietnam. Our findings revealed that treating sleep deficiency among older adults in the Philippines and Vietnam can potentially reduce the risks of multimorbidity.

